# Characterization of hypersensitivity reactions reported among *Andrographis paniculata* users in Thailand using Health Product Vigilance Center (HPVC) database

**DOI:** 10.1186/1472-6882-14-515

**Published:** 2014-12-24

**Authors:** Wimon Suwankesawong, Surasak Saokaew, Unchalee Permsuwan, Nathorn Chaiyakunapruk

**Affiliations:** Health Product Vigilance Center (HPVC), Food and Drug Administration, Ministry of Public Health, Bangkok, Thailand; Center of Health Outcomes Research and Therapeutic Safety (COHORTS), School of Pharmaceutical Sciences, University of Phayao, Phayao, Thailand; Center of Pharmaceutical Outcomes Research (CPOR), Department of Pharmacy Practice, Faculty of Pharmaceutical Sciences, Naresuan University, Phitsanulok, Thailand; Pharmacotherapy Outcomes Research Center (PORC), College of Pharmacy, University of Utah, Salt Lake City, UT USA; Department of Pharmaceutical Care, Faculty of Pharmacy, Chiang Mai University, Chiang Mai, Thailand; School of Pharmacy, Monash University Malaysia, Selangor, Malaysia; School of Population Health, University of Queensland, Brisbane, QLD Australia; School of Pharmacy, University of Wisconsin-Madison, Madison, WI USA

**Keywords:** *Andrographis paniculata*, Database, Health Product Vigilance Center (HPVC), Hypersensitivity Reactions, Thailand

## Abstract

**Background:**

*Andrographis paniculata* (andrographis) is one of the herbal products that are widely used for various indications. Hypersensitivity reactions have been reported among subjects receiving *Andrographis paniculata* in Thailand. Understanding of characteristics of patients, adverse events, and clinical outcomes is essential for ensuring population safety.

This study aimed to describe the characteristics of hypersensitivity reactions reported in patients receiving andrographis containing products in Thailand using national pharmacovigilance database.

**Methods:**

Thai Vigibase data from February 2001 to December 2012 involving andrographis products were used. This database includes the reports submitted through the spontaneous reporting system and intensive monitoring programmes. The database contained patient characteristic, adverse events associated with andrographis products, and details on seriousness, causality, and clinical outcomes. Case reports were included for final analysis if they met the inclusion criteria; 1) reports with andrographis being the only suspected cause, 2) reports with terms consistent with the constellation of hypersensitivity reactions, and 3) reports with terms considered critical terms according to WHO criteria. Descriptive statistics were used.

**Results:**

A total of 248 case reports of andrographis-associated adverse events were identified. Only 106 case reports specified andrographis herbal product as the only suspected drug and reported at least one term consistent with constellation of hypersensitivity reactions.

Most case reports (89%) came from spontaneous reporting system with no previously documented history of drug allergy (88%). Of these, 18 case reports were classified as serious with 16 cases requiring hospitalization. For final assessment, the case reports with terms consistent with constellation of hypersensitivity reactions and critical terms were included. Thirteen case reports met such criteria including anaphylactic shock (n = 5), anaphylactic reaction (n = 4) and angioedema (n = 4). Time to development of symptoms ranged from 5 minutes to 1 day. The doses of andrographis used varied from 352 mg to 1,750 mg. Causality assessment of 13 case reports were certain (n = 3), probable (n = 8) and possible (n = 2).

**Conclusions:**

Our findings suggested that hypersensitivity reactions have been reported among patients receiving *Andrographis paniculata*. Healthcare professionals should be aware of this potential risk. Further investigation of the causal relationship is needed; meanwhile including hypersensitivity reactions for andrographis product labeling should be considered.

## Background

*Andrographis paniculata* (andrographis) is one of the herbal products that have been widely used for a variety of medical purposes. It is widely found and cultivated in tropical and subtropical Asia, South-East Asia, and India. The leave part has been used traditionally to treat fever, and infection especially those related to respiratory tract infections [1]. A systematic review of safety and efficacy of andrographis from seven double-blinded, controlled trials indicated a superiority in alleviating the subjective symptoms of uncomplicated upper respiratory tract infection of andrographis compared with placebo[1]. Mild and infrequent adverse events were reported following its use [1]. However, the interpretation of safety data should be cautious from efficacy trials due to of the absence of rigorous data collection on adverse events [1]. Additionally, the Uppsala Monitoring Centre (UMC) which maintains the WHO international pharmacovigilance programme in collaboration with other 112 countries around the world collected 19 case reports of suspected adverse reactions from andrographis containing oral products [2]. Of those, 17 reports concerned acute hypersensitivity reactions which included 7 reports of anaphylaxis. Even though andrographis product is often perceived as a safe herbal product with minor adverse effects, it is recommended that a warning be included in the product information of andrographis containing oral products [2].

In Thailand, a single ingredient product of andrographis in oral preparation forms such as capsule, tablet and bolus has been listed in the National List of Essential Medicines (NLEM) for use in the indication of non-infectious diarrhea and pharyngotonsillitis since 1999 [3]. Its indication has been expanded to common cold since 2006 [4, 5]. To ensure its safety, an intensive monitoring programme was developed as a part of NLEM standard process in order to surveillance for adverse events that might occur. The program was responsible by The Health Product Vigilance Center (HPVC), which was established under the Thai Food and Drug Administration, Ministry of Public Health in 1983 (http://thaihpvc.fda.moph.go.th). Under this intensive monitoring programme, 1,873 patients using andrographis oral containing products (199 during 2001–2003, and 1,674 during 2007–2009) were reported that andrographis could possibly cause various adverse events such as rash, pruritus, breath discomfort, cough, peeling skin, fatigue, nausea, gastric discomfort, anorexia, headache and palpitation [6, 7]. It should be noted that direct causation has not been substantiated and other contributing factors could be playing a role in adverse events being reported. After this period, the HPVC has continued monitoring the safety of this product through spontaneous reporting system. Despite some limitations such as under-reporting and limited quality of reports, the spontaneous reporting system is still a valuable mechanism in identifying adverse events and their characteristics which may lead to regulatory actions to improve patient safety [8–11].

Thai Vigibase has been developed by the HPVC as the Thai national database collecting all case reports submitted from the spontaneous reporting system or intensive monitoring programmes [12]. Intensive monitoring programmes were undertaken to promote the reporting of adverse events associated with herbal product use. After a total of five single herbal products were included in the Thai NLEM in 1999, a 2-year intensive monitoring programme of these products was launched in the year 2000. In addition, an intensive monitoring programme was initiated by the Department for Development of Thai Traditional and Alternative Medicine (which is responsible for promoting the use of herbal products in Thailand). This programme focused on four single herbal products (*Cissus quadrangularis* [Veld grape], *Centella asiatica* [Pennywort], *Derris scandens* [Jewel vine], *Momordica charantia* [Bitter melon]) that were under consideration for inclusion in the NLEM. Data collected under this programme will be crucial information used for considering whether these products are to be listed in NLEM. Suspected adverse events have been reported by healthcare professionals from more than 900 health facilities in Thailand to the HPVC. The submission process can be either via an adverse event reporting form or via an online reporting system. Currently, Thai Vigibase has contained over 500,000 reports [13]. Until now, there have been only two published studies [14, 15] using Thai Vigibase. Since Thai Vigibase has been considered a valuable source to look into adverse event reports among herbal products [14], characterizing patients and adverse events associated with andrographis can provide insights into a better understanding of this product safety profile. This retrospective database study aimed to describe characteristics of patients and hypersensitivity reactions associated with andrographis containing products using the Thai Vigibase database.

## Methods

### Data source

This retrospective database study used the Thai Vigibase which included all case reports of suspected adverse events submitted by healthcare professionals throughout the country to HPVC from February 2001 to December 2012. The case reports were obtained through either the spontaneous reporting system or intensive monitoring programmes [12]. Since this is an aggregate analysis of spontaneous reports of adverse events submitted to HPVC, there is minimal risk of patient confidentiality breaching. As we did not obtain the consent, we present specific cases with only general demographic characteristics to ensure patient identity anonymous. Permission for database access and publication of this work was granted by Thai FDA.

### Criteria for the selection of cases

Reports with andrographis being the only suspected cause were selected. To be specific in final analysis, we investigated in details only those reports with terms consistent with constellation of hypersensitivity reactions [16] and considered critical terms according to WHO criteria [17].

### Data extraction

All reports that indicated andrographis as the only suspected drug were retrieved. Of those reports, only ones that represented at least one hypersensitivity reaction[16] based on inclusion criteria were extracted. Finally, we matched hypersensitivity reaction with the WHO Adverse Reaction Terminology (WHO-ART) classification. According to WHO-ART classification, each preferred term is categorized into a primary system organ class and up to two subsidiary system organ classes [17]. We used the matched pair of the primary system organ class and preferred term in our analysis. A unique HPVC number identified each unique report. From each report, the following information was extracted: patient demographics, co-morbidities, suspected reactions, suspected drug as well as concomitant treatments, dosage, duration of andrographis treatment, date of adverse event onset, clinical outcome, causality assessment, and the source of reports. The seriousness of adverse event was classified as non-serious and serious events. For serious adverse events, they were sub-classified into hospitalization or prolongation of hospitalization, persistent or significant disability/incapacity, and life threatening. Data in Thai Vigibase were in the Oracle® format.

### Data analyses

The data were managed using Microsoft Excel® and subsequently imported into STATA® version 12.0 (StataCorp, College Station, Texas) for analysis. All variables were analyzed using descriptive statistics to determine total number of reports; mean age and number in each age group; percentage of gender, history of drug allergy, distribution of adverse events, source of reports, causality assessment, time of exposure, number of concomitant treatments, and time to onset of reaction.

## Results

**Figure 1:**
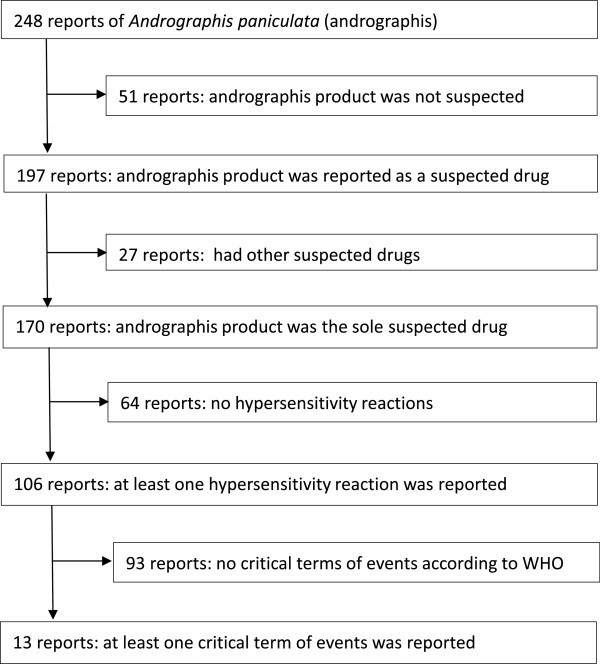
**Flow diagram for case reports selection.**

A total of 197 case reports of adverse events associated with *Andrographis paniculata* containing products were retrieved. In all reports, the herbal product was taken by mouth and was sole suspected drug in 170 case reports. A total of 106 cases reported term (s) consistent with constellation of hypersensitivity reactions (Figure 1).

Characteristics of 106 cases were provided in Table 1. Most of them (89.6%) came from spontaneous reporting system. Age ranged from 8 – 80 years with a mean age of 38.5 ± 18.4 years old and 71.7% were female. Almost half of them (46.2%) were in 31–60 years age group. Eighty-seven percent had no previously documented history of drug allergy. Only 17% (18 cases) were classified as serious with 16 cases requiring hospitalization. None of these resulted in death. For the causal relationship of reported event and the suspected herbal drugs, 57.6% were assessed as probable, followed by possible 31.1% and certain 7.5%. Most of them (77.4%) were no concomitant drug. Forty-eighth percent had time of exposure less than 1 day. The reactions had developed in the first day of administration in 57.6% of case reports and 22.6% on the second days of use. The reported adverse events were shown in Table 2.

**Table 1:** **Characteristics of patients and adverse event reports among andrographis products users**

Characteristics	No. (%) [n = 106] ^a^
**Patients**	
Sex	
Female	76 (71.7)
Male	30 (28.3)
Age [y], mean (SD) [min – max]	38.5 (18.4) [8 – 80]
<15	11 (10.4)
15-30	26 (24.5)
31-60	49 (46.2)
>60	11 (10.4)
N/A	9 (8.5)
History of drug allergy	
No	93 (87.7)
Yes	12 (11.3)
N/A	1 (1.0)
**Adverse event reports**	
Source of reports	
Spontaneous	95 (89.6)
Intensive	11 (10.4)
Seriousness	
Serious	18 (17.0)
Hospitalization or prolongation of hospitalization	16
Life threatening	2
Non-serious	88 (83.0)
Causality assessment^b^	
Certain	8 (7.5)
Probable	61 (57.6)
Possible	33 (31.1)
Unlikely	0 (0.0)
N/A	4 (3.8)
No. of concomitant drug, mean (SD) [min – max]	0.4 (0.8) [0 – 4]
None	82 (77.4)
1item	13 (12.3)
2items	7 (6.6)
3items	3 (2.8)
≥4items	1 (0.9)
Time of exposure (day), mean (SD) [min – max]	1.2 (3.4) [0 – 30]
1 day	51 (48.1)
2 days	20 (18.9)
3 days	14 (13.2)
≥4 days	8 (7.6)
N/A^c^	13 (12.2)
Time to onset (day), mean (SD) [min – max]	0.8 (1.6) [0 – 8]
First day of use	61 (57.6)
2daysof use	24 (22.6)
3 days of use	15 (14.2)
≥4 days of use	5 (4.7)
N/A	1 (0.9)

^a^All 106 reports contained hypersensitivity reactions and an andrographis product was considered as the sole suspected drug.

^b^As per reported from health professional.

^c^Some reports had no information on starting date, stopping date, or both.

**N/A** = not available.

**Table 2:** **Adverse events classified by organ system according to WHO Adverse Reaction Terminology**

System organ class	No. (%) [n = 243] ^a^	Preferred term(n)
Skin and appendages disorders	126(51.85)	Urticaria (37), rash maculo-papular (31), rash (18), pruritus (16), rash erythematous (8), dermatitis exfoliative (1), skin exfoliation (2), Steven Johnson syndrome (1), sweating increased (2), acute generalized exanthematous pustulosis (1), bullous eruption (1), eosinophillia (1), fixed eruption (1), stomatitis ulcerative (1), purpura allergic (1), flushing (1)
Body as a whole-general disorders	57(23.46)	Face oedema (19), **angioedema (4)**, **anaphylactic shock(5), anaphylactic reaction (4),** fatigue (6), oedema mouth (5), fever (3), chest pain (3), oedema (2), oedema periorbital (1), therapeutic response decreased (2), back pain (1), oedema peripheral(3), pain(1)
Gastrointestinal system disorders	22(9.05)	Nausea (8), vomiting (4), abdominal pain (2), diarrhea (1), gastro-intestinal disorder non-specific (1), lipsdry (1), stomatitis (1), melaena (1), mouth dry (1)
Respiratory system disorders	15(6.17)	Dyspnoea (6), coughing (6), bronchospasm (2), sputum increased (1)
Psychiatric disorders	8(3.29)	Anorexia (4), somnolence (3), insomnia(1)
Central and peripheral nervous system disorders	7(2.88)	Headache (3), dysaethesia (2), dizziness(2)
Musculoskeleton system disorders	3(1.23)	Muscle weakness (2), paralysis (1)
Vascular (extra cardiac) disorders	1(0.41)	Vasculitis (1)
Liver and biliary system disorders	1(0.41)	Hepatitis(1)
Urinary system disorders	1(0.41)	Urinary frequency (1)
Various	2(0.82)	Anaesthesia local (1), anaesthesia mouth(1)

^a^Number of events from 106 case reports (some reports have more than one event).

Bold indicates hypersensitivity reactions with critical term.

Out of 106 case reports, a total of 13 case reports were included for detail case description since their reactions were hypersensitivity reactions considered critical term according to WHO criteria. Adverse events included anaphylactic shock (5), anaphylactic reaction (4) and angioedema (5). The average age was 38.9 years old. Most of them (10/13, 76.9%) were female. Details of each case including adverse event reports were shown in Table 3. A history of drug allergy was present in two patients who developed angioedema. One patient was allergic to tetracycline, while another one was allergic to ibuprofen. The onset ranged from 5 minutes to 1 day. Most of them (11 case reports) were serious. The probability of causality assessment was certain (3), probable (7) and possible (1). Of 13 case reports, 7 case reports indicated the product’s brand name. Most importantly, 6 brands were reported among the 7 case reports. After checking each brand name, all of them are dry powder with different doses ranging from 352 mg to 1,750 mg.

**Table 3:** **Hypersensitivity reactions with critical term among patients using andrographis product**

Case no.	Product			Adverse event/symptom					Outcome
	Drug	Type ^a^	Dosage	Adverse event	Details	Onset	Seriousness	Causality assessment	
1	andrographis (325 mg/cap)	S	5 cap PO, OD	anaphylactic shock	Angioedema, urticaria, BP 90/60	30 min	serious (hospitalization or prolongation of hospitalization)	Certain	Complete recovery without lesion
2	andrographis (352 mg/cap)	S	3 cap 1 time used	anaphylactic shock	Urticaria, BP 87/73	1 day	serious (hospitalization or prolongation of hospitalization)	Probable	Complete recovery without lesion
	paracetamol (500 mg)	O	1 tab PO, OD						
	chlorpheniramine (4 mg)	O	1 tab PO, TID						
3	andrographis	S	2 cap 1 time used	anaphylactic shock	Urticaria, BP 90/70	30 min	serious (hospitalization or prolongation of hospitalization)	Probable	Complete recovery without lesion
	paracetamol (500 mg)	O	2 tab PO, prn						
4	andrographis	S	2 cap 1 time used	anaphylactic shock	Oedema, uticaria, chest pain, BP 130/70	1 day	serious (hospitalization or prolongation of hospitalization)	Probable	Complete recovery without lesion
5	andrographis (352 mg/cap)	S	1 cap PO, TID	anaphylactic shock	Puffy eyelid, lung wheezing, drowsiness, BP 80/50	1 day	serious (life threatening)	Certain	Complete recovery without lesion
6	andrographis	S	1750 mg 1 time used	anaphylactic reaction	Urticaria, sweat increased, nausea, BP 96/59	10 min	serious (hospitalization or prolongation of hospitalization)	Probable	Complete recovery without lesion
7	andrographis	S	350 mg PO, 1 time used	anaphylactic reaction	anaphylactic reaction, BP 120/80	45 min	serious (life threatening)	Probable	Complete recovery without lesion
8	andrographis	S	400 mg 2 time used	anaphylactic reaction	Oedema eyelid, wheezing, rash, BP 153/101	16 hr	serious (hospitalization or prolongation of hospitalization)	Probable	Complete recovery without lesion
9	andrographis	S	2 cap PO, 1 time used	anaphylactic reaction	Urticaria, rash, chest tightness, , tachycardia	5 min	serious (hospitalization or prolongation of hospitalization)	Probable	Complete recovery without lesion
10	andrographis	S	4 cap PO, TID	angioedema	Angioedema, urticaria	1 day	Non-serious	Certain	Complete recovery without lesion
11	andrographis	S	3 cap PO, TID	angioedema	-	1 day	Non-serious	Possible	Complete recovery without lesion
	guaifenesin (100 mg)	O	1 tab PO, TID						
	Salbutamol (4 mg)	O	1 tab PO, TID						
12	andrographis	S	N/A	angioedema	-	N/A	Non-serious	Possible	Complete recovery without lesion
	ranitidine	O	N/A						
	hydrochlorothiazide	O	N/A						
	*Curcuma longa* Linn.	O	N/A						
13	andrographis	S	N/A	angioedema	-	1 day	Non-serious	Probable	Still has some symptom

^a^S = Suspected product, O = Other product.

*Abbreviations*: *PO* Per oral, *OD* once a day, *TID* three times a day, *QID* Quater In Die (four times a day), *prn* Pro Re Nata (as needed), *N/A* not available, *min* minute (s), *hr* hour (s).

## Discussion

Our study is the first report accumulating a number of case reports with hypersensitivity reactions especially anaphylactic reactions among andrographis users in Thailand. Our findings indicated that the oral use of andrographis may be associated with a risk of acute hypersensitivity reactions. However, it was not possible to draw a direct causal relation from this study. There remains a strong need warranting a further study to investigate this association in the future.

Even though andrographis had long been used as traditional medicines particularly in Southeast Asia, e.g. India, Thailand, there has been limited report about hypersensitivity reaction associated with andrographis. To the best of our search, there was only one study [18] reporting a case of anaphylactic reaction among an HIV patient. This study was conducted in 13 HIV positive patients and 5 uninfected health-volunteer to evaluate adverse events of escalating dosages. At the elevated dose of andrographis (6–12 times higher than usual dose), a case of HIV patient experienced an anaphylactic reaction during week 4 and stopped medication. It is not uncommon to expect such adverse event when any particular product is given in such circumstance. It was important to note that there was no mention on the mechanism of such hypersensitivity reaction.

The number of cases reports of hypersensitivity analysis among andrographis users in this study was 106, which was much higher than 17 cases reported in the first document describing the international experiences in pharmacovigilance network [2]. In addition, these reports were submitted from various settings with differing brand products. This cumulative evidence suggested that andrographis was potentially associated with hypersensitive reactions as it was the likely cause for the majority of cases.

It is important to note that all case reports in this study used andrographis containing product as a single agent. This is very different from others [2]. Farah et al. [2] described 17 reports of hypersensitivity reactions including 7 anaphylaxis, and specified that the case reports consist of both single agent and combination of agents in products containing andrographis. It was suggested that the use andrographis as a single agent or purified form as andrographolide should be prohibited. Calabrese et al. [18] hypothesised that this herb has been used in combination with other herbs, perhaps to ameliorate potential adverse effects from andrographis.

The issue of reporting of hypersensitivity reactions among andrographis users has caught attention of Thai FDA. The Signal Detection and Assessment Working Group and Drug Safety (pharmacovigilance) subcommittee under Thai FDA decided to make no changes on product labelling because the causation could not be confirmed. The report of potential association was disseminated through HPVC (Health Products Vigilance Center) safety news. In addition, Thai FDA recommends a pharmacoepidemiology study to determine whether the association between hypersensitivity reactions and andrographis containing products is confirmed.

An important limitation of current evidence is the lack of direct causation. Our study cannot eliminate a possibility that hypersensitivity reaction might be related to product contamination and its lack of standardization across brands. Even though the evidence presented in this article is substantiated with a large number of case reports submitted from various settings with differing brand products, there remains no direct causation study that can make such a conclusive remark.

## Conclusion

In summary, our findings demonstrated that hypersensitivity reactions have been reported among patients receiving *Andrographis paniculata*. Healthcare professionals should be aware of this potential risk. Further investigation of the causal relationship is needed.
